# Determining the Outcomes of Pilon Fractures Treated by Minimally Invasive Plate Osteosynthesis (MIPO): A Retrospective Study

**DOI:** 10.7759/cureus.91587

**Published:** 2025-09-04

**Authors:** Shobhet Saxena, Nagakumar J S, Hariprasad Seenappa, Punith K, Tarun Kumar Somisetty Venkata Sai, Ayush Agrawal

**Affiliations:** 1 Orthopaedics, Sri Devaraj Urs Academy of Higher Education and Research, Kolar, IND; 2 Orthopaedics, RL Jalappa Hospital and Research Centre, Kolar, IND

**Keywords:** fracture reduction, nonunion, open reduction and internal fixation, osteosynthesis, pilon fracture

## Abstract

Background: Pilon fractures, affecting the distal tibia, pose significant challenges due to their complexity and the delicate balance required between adequate fracture reduction and soft tissue preservation. Minimally invasive plate osteosynthesis (MIPO) has emerged as a viable alternative to traditional open reduction and internal fixation (ORIF), aiming to reduce soft tissue complications while maintaining biomechanical stability.

Objective: This study evaluates the functional and radiological outcomes of pilon fractures treated via MIPO, analyzes correlations between radiographic union and clinical scores, and investigates complication rates.

Methods: We retrospectively analyzed 33 patients with AO/OTA (Arbeitsgemeinschaft für Osteosynthesefragen/Orthopaedic Trauma Association) 43-B and 43-C pilon fractures treated using MIPO from February 2023 to January 2024 at RL Jalappa Hospital and Research Centre, Kolar, India. Functional outcomes were measured using the American Orthopaedic Foot & Ankle Society Hindfoot Score (AOFAS), Olerud and Molander Ankle Score (OMAS), and visual analog scale (VAS) scores. Radiological union was assessed using the modified Radiographic Union Scale for Tibial fractures (mRUST). Statistical analyses included Kruskal-Wallis, Mann-Whitney U, chi-square, and Fisher’s exact tests.

Results: Results suggest favorable functional recovery (mean AOFAS score: 85.2 ± 8.6) and a low complication rate (9.1%, n=3, N=33). Radiological union was achieved in 93.9% (n=31, N=33) of cases by six months.

Conclusion: MIPO demonstrates favorable functional and radiological outcomes in pilon fractures with reduced soft tissue complications. Further multicentric studies are warranted to validate long-term efficacy.

## Introduction

Pilon fractures, also referred to as tibial plafond fractures, are complex injuries involving the distal metaphysis and articular surface of the tibia. These fractures are predominantly caused by high-energy axial loading mechanisms, such as falls from height or motor vehicle accidents, and are often associated with significant soft tissue damage, articular comminution, and metaphyseal-diaphyseal dissociation. They account for approximately 7-10% of all tibial fractures, and their management remains one of the most challenging tasks in orthopaedic trauma care [1].

The primary goal of treatment in pilon fractures is to restore the congruity of the articular surface while preserving the surrounding soft tissue envelope. Traditional approaches have predominantly relied on open reduction and internal fixation (ORIF), which allows for accurate reduction and rigid fixation. However, this technique requires extensive surgical dissection, which increases the risk of postoperative complications, particularly in cases where the soft tissue is already compromised. Reports indicate that wound complications, including infection, dehiscence, and necrosis, may occur in up to 55% of cases managed via ORIF [2].

To address these complications, less invasive methods such as minimally invasive plate osteosynthesis (MIPO) have been introduced [3]. MIPO utilizes small incisions, indirect reduction techniques, and submuscular plate insertion to minimize surgical trauma and preserve the periosteal blood supply [4]. This approach is grounded in the principles of biological fixation, which favor relative stability and soft tissue preservation over anatomical reduction alone. Several studies have suggested that MIPO offers comparable or even superior outcomes to ORIF in terms of union rates, functional recovery, and complication profiles [5,6].

Despite promising results, there remains a paucity of comprehensive data on the long-term clinical and radiological outcomes of pilon fractures treated via MIPO, particularly in resource-limited settings. Moreover, the correlation between radiographic healing scores and functional outcomes, such as pain and mobility, has not been fully elucidated. Studies also vary in defining the optimal fracture patterns suitable for MIPO, with some suggesting better outcomes in simpler fracture types such as AO/OTA (Arbeitsgemeinschaft für Osteosynthesefragen/Orthopaedic Trauma Association) 43-B compared to more comminuted 43-C variants [7-10].

Another consideration in the treatment of pilon fractures is the timing of surgery. A staged protocol - initial stabilization with spanning external fixation, followed by delayed definitive fixation - is now commonly advocated to allow soft tissue recovery prior to plate application [3,11]. This approach has been associated with a reduction in wound complications and better overall outcomes [11,12].

Given this context, our retrospective study was designed to evaluate both the functional and radiological outcomes of pilon fractures treated with MIPO over a one-year period. Specifically, we aimed to assess the extent to which MIPO achieves fracture union, reduces pain, and restores function, as measured by validated scoring systems [13,14]. We also sought to identify any differences in outcomes between fracture subtypes (43-B vs 43-C) and to examine the incidence and nature of complications following this surgical technique [15]. By addressing these clinical questions, our study contributes to the growing body of evidence supporting MIPO as a safe and effective alternative to ORIF for selected pilon fractures.

## Materials and methods

Study design and setting

This retrospective study was conducted at RL Jalappa Hospital and Research Centre, Tamaka, Kolar, India, from February 2023 to January 2024, after obtaining institutional ethical clearance.

A total of 33 adult patients (≥18 years) with closed AO/OTA 43-B and 43-C pilon fractures treated with MIPO were considered for the study by using the inclusion and exclusion criteria. Inclusion and exclusion criteria have been mentioned in Table 1.

**Table 1:** Inclusion and exclusion criteria AO/OTA: Arbeitsgemeinschaft für Osteosynthesefragen/Orthopaedic Trauma Association

Category	Criteria
Inclusion	Age ≥18 years
	AO/OTA 43-B and 43-C fractures
	Treated with MIPO with one-year follow-up, with complete radiographic and functional data
Exclusion	Pathological fractures
	Prior ipsilateral ankle injury/surgery

Surgical technique

All procedures were performed using MIPO through small incisions, under fluoroscopic guidance, with a pre-contoured distal tibial locking plate. Patients underwent staged protocols where needed, with initial spanning external fixation, followed by definitive fixation once soft tissues were favorable.

Data collection

Data were retrieved from medical records using standardized formats. Collected variables included socio-demographic information such as age, sex, and body mass index (BMI); clinical parameters such as comorbidities, medications usage, and previous illnesses; intraoperative details such as time of surgery, approach, and blood loss; postoperative data such as duration of hospital stay and complications; functional outcomes, including American Orthopaedic Foot & Ankle Society Hindfoot Score (AOFAS), Olerud and Molander Ankle Score (OMAS), and visual analog scale (VAS) at 12 months; and radiological outcomes, including modified Radiographic Union Scale for Tibial fracture (mRUST) scores at 3, 6, 9, and 12 months.

Outcome measures

The following were the outcome measures used: the AOFAS Hindfoot Score, which evaluates pain, function, and alignment (range: 0-100); OMAS, which is an ankle-specific functional score (range: 0-100); VAS, which is a scale for pain intensity (0-10); and mRUST, which is a radiological healing score (range: 4-12), assessing cortical healing on standard radiographs.

Statistical analysis

Statistical analysis was performed using Statistical Product and Service Solutions (SPSS, version 22.0; IBM SPSS Statistics for Windows, Armonk, NY). Normality was assessed via the Kolmogorov-Smirnov test. Kruskal-Wallis and Mann-Whitney U tests were applied for continuous non-parametric variables. Chi-square and Fisher's exact tests were used for categorical variables. Significance was set at p<0.05.

## Results

As shown in Table 2, the mean age of study participants was 42.5 ± 12.3 years, with a gender distribution of 26 (78.8%, N=33) males and seven (21.2%, N=33) females. Fractures were classified as 43-B in 18 (54.5%, N=33) cases and 43-C in 15 study participants (45.5%, N=33). Functional outcomes were assessed using the AOFAS score, and it is presented in Table 3. It showed a mean of 85.2 ± 8.6 (range: 62-98), with excellent or good outcomes achieved in 27 (81.8%, N=33) patients. The OMAS mean score was 78.4 ± 9.2 (range: 55-95), while the VAS pain score demonstrated significant improvement from a preoperative mean of 7.8 ± 1.2 to 1.5 ± 1.1 at one year postoperatively (p<0.001). As presented in Table 4, radiological outcomes, evaluated using the mRUST score, indicated progressive fracture healing, with scores of 7.2 ± 1.5 at six weeks, 10.8 ± 1.3 at 12 weeks, and 12.0 ± 0.0 at 24 weeks, with full union achieved in 31 cases (93.9%, N=33). Postoperative complications were minimal, including superficial infection in two (6.1%, N=33) patients and delayed union in one (3.0%, N=33) patient, with no reported cases of nonunion or implant failure, as presented in Table 5. Table 6 explores the relationship between fracture type (43-B and 43-C) and clinical outcomes, including functional recovery, healing time, and complications. Patients with 43-B fractures demonstrated significantly better functional outcomes, as measured by the AOFAS score (88.3 ± 6.2), compared to those with 43-C fractures (AOFAS score: 81.5 ± 9.8), with a statistically significant difference (p=0.02). Additionally, 43-B fractures healed faster, with an average union time of 18.1 ± 3.2 weeks, whereas 43-C fractures took significantly longer (22.4 ± 4.1 weeks; p=0.01). While the 43-C group had a higher complication rate (13.3%, n=2, N=33) compared to the 43-B group (5.6%, n=1, N=33), this difference was not statistically significant (p=0.42), possibly due to the limited sample size. The findings suggest that 43-C fractures, being more severe, are associated with poorer functional recovery and delayed healing compared to 43-B fractures, though complication rates did not differ significantly in this study. Further research with larger cohorts may help clarify the risk of complications between these fracture types.

**Table 2:** Demographic and fracture characteristics of the study participants *Age, BMI: Mean ± Standard Deviation

Variable	Value (n=33)
Age (years)	42.5 ± 12.3 (18–65)*
Gender	
Male	26 (78.8%)
Female	7 (21.2%)
Fracture Type (AO/OTA)	
43-B	18 (54.5%)
43-C	15 (45.5%)
BMI (kg/m²)	26.4 ± 3.8*
Mechanism of Injury	
Fall from height	14 (42.4%)
Motor vehicle accident	19 (57.6%)

**Table 3:** Functional outcomes at one-year follow-up AOFAS: American Orthopaedic Foot & Ankle Society Hindfoot Score, OMAS: Olerud and Molander Ankle Score, VAS: visual analog scale

Outcome Measure	Mean ± SD	Range
AOFAS Score	85.2 ± 8.6	62–98
OMAS	78.4 ± 9.2	55–95
VAS Pain Score		
Preoperative	7.8 ± 1.2	6–10
Postoperative	1.5 ± 1.1	0–

**Table 4:** Radiological union (mRUST score progression) among the study participants mRUST: modified Radiographic Union Scale for Tibial fractures

Time Point	Mean mRUST ± SD	Union Rate (N=33)
6 weeks	7.2 ± 1.5	12.1%
12 weeks	10.8 ± 1.3	72.7%
24 weeks	12.0 ± 0.0	93.9%

**Table 5:** Postoperative complication distribution among the study participants

Complication	Frequency (N=33)	Percentage
Superficial infection	2	6.1%
Delayed union	1	3.0%
Nonunion	0	0%
Implant failure	0	0%

**Table 6:** Correlation between fracture type and outcomes χ² degree of freedom = 1, Statistically significant: p < 0.05* AOFAS: American Orthopaedic Foot & Ankle Society Hindfoot Score

Variable	43-B (n=18)	43-C (n=15)	p-value	Statistical Test	Effect Size
AOFAS score	88.3 ± 6.2	81.5 ± 9.8	0.02*	Mann-Whitney U = 75.5	r = 0.41 (medium)
Union time (weeks)	18.1 ± 3.2	22.4 ± 4.1	0.01*	Mann-Whitney U = 62.0	r = 0.48 (large)
Complication rate	5.6% (n=1)	13.3% (n=2)	0.42	χ² = 0.65 (Fisher's exact test)	Cramer's V = 0.14 (small)

## Discussion

The findings of this study show the efficacy of MIPO in the management of pilon fractures, particularly in terms of functional recovery, radiological union, and complication rates. The results align with and expand upon existing literature, providing further evidence for MIPO as a viable alternative to traditional ORIF.

Functional recovery

The mean AOFAS score of 85.2 ± 8.6 and OMAS score of 78.4 ± 9.2 at the 12-month follow-up indicate that the majority of patients achieved excellent or good functional outcomes. These results are consistent with prior studies, such as Barış et al. [6], who reported similar functional improvements with MIPO [6]. The significant reduction in VAS pain scores (from 7.8 preoperatively to 1.5 postoperatively) further highlights the clinical benefits of MIPO, including reduced postoperative pain and enhanced early mobilization. The preservation of soft tissue integrity and periosteal blood supply likely contributed to these favorable outcomes, as MIPO minimizes surgical trauma compared to ORIF.

Radiological union

The high radiological union rate (93.9% by 24 weeks, n=31, N=33) and the progressive improvement in mRUST scores (from 7.2 at six weeks to 12.0 at 24 weeks) demonstrate the biomechanical stability of MIPO. These findings support the work of Gao et al. [5], who emphasized the importance of stable fixation in achieving consistent fracture healing [5]. Notably, the absence of significant malalignment in our cohort suggests that MIPO can effectively maintain anatomical reduction while reducing the risk of secondary displacement - a critical advantage in complex pilon fractures.

Complication profile

The low complication rate (9.1% overall, with only 6.1% superficial infections and 3.0% delayed union, N=33) contrasts sharply with the higher rates reported in ORIF studies, such as those by Teeny et al., where wound complications approached 55% [2]. This stark difference underscores MIPO's superiority in preserving soft tissue viability, particularly in high-energy injuries where soft tissue compromise is common. The absence of nonunion or implant failure in our study further reinforces the reliability of MIPO as a surgical technique.

Comparative analysis of fracture types

Our subgroup analysis revealed that AO/OTA 43-B fractures had significantly better functional outcomes (mean AOFAS: 88.3 vs. 81.5 for 43-C) and shorter union times (18.1 vs. 22.4 weeks) compared to 43-C fractures. This suggests that, while MIPO is effective for both fracture types, its benefits may be more pronounced in less severe (43-B) fractures. However, the small sample size limits the generalizability of these findings, warranting further investigation.

Limitations

Despite the promising results, this study has several limitations. This study has a retrospective design and may be susceptible to selection bias, as patients were not randomized. With a small sample size, with only 33 patients, the statistical power is limited, particularly for subgroup analyses. As there is no control group, the absence of a comparative ORIF or external fixation cohort restricts direct comparisons. The follow-up duration is short. A 12-month follow-up is insufficient to assess long-term complications, such as post-traumatic arthritis, which may develop years after the injury.

## Conclusions

This study demonstrates that MIPO is a safe and effective treatment for pilon fractures, offering excellent functional recovery, high union rates, and minimal complications. The technique's ability to preserve soft tissue integrity and reduce surgical trauma makes it particularly advantageous in high-energy injuries where soft tissue compromise is a concern. While ORIF remains a valuable option in certain scenarios, MIPO should be strongly considered for AO/OTA 43-B and 43-C fractures, especially in patients with compromised soft tissues. This study adds to the growing body of evidence supporting MIPO as a preferred surgical approach for pilon fractures, balancing biomechanical stability with minimal soft tissue disruption. To further validate these findings, prospective, multicenter studies with larger cohorts and longer follow-up periods are needed. Future studies should aim to optimize patient selection and surgical techniques to further improve outcomes.
